# Raw fastq data for hotspot regions of cancer-related 50 genes using fresh frozen breast carcinoma tissues obtained from IMERI-FMUI biobank collections

**DOI:** 10.3389/fgene.2022.973453

**Published:** 2022-10-24

**Authors:** Ria Kodariah, Fadilah Fadilah, Rafika Indah Paramita, Linda Erlina, Khaerunnisa Anbar Istiadi, Yayi Dwina Billianti, Meilania Saraswati, Sonar Soni Panigoro

**Affiliations:** ^1^ Master’s Programme in Biomedical Sciences, Faculty of Medicine, Universitas Indonesia, Jakarta, Indonesia; ^2^ Department of Anatomical Pathology, Faculty of Medicine, Universitas Indonesia, Jakarta, Indonesia; ^3^ Department of Medical Chemistry, Faculty of Medicine, Universitas Indonesia, Jakarta, Indonesia; ^4^ Bioinformatics Core Facilities-IMERI, Faculty of Medicine, Universitas Indonesia, Jakarta, Indonesia; ^5^ Department of Biology, Institut Teknologi Sumatera, Lampung, Indonesia; ^6^ Surgical Oncology Division, Department of Surgery, Faculty of Medicine, Universitas Indonesia, Jakarta, Indonesia

**Keywords:** biobank IMERI-FMUI, breast cancer, frozen tissues, hot-spot gene sequencing, NGS–next generation sequencing

## 1 Introduction

The creation of biobank has steadily developed additional research platforms and created opportunities over the years to learn more about how living systems function in both acute and chronic physiological and pathological circumstances. It involves a process of gathering, preserving, distributing, and utilization of biological samples for prospective research studies. The majority of hospitals and biomedical research facilities in many countries, in Indonesia as well, participate in this activity, which is essential to the development of a successful, effective, and cutting-edge research system. Potential in the new biology era could create fascinating possibilities for comprehending the physiological and pathological mechanisms behind human health by unravelling the more intricate processes (Caenazzo and Tozz, 2020).

Early population-based biobanks generally focused on finding genetic variations linked to disease without taking into account how the information may be relayed back to participants for their own health management. The genetic basis of illness susceptibility varies between ethnicities since many disease-causing variants are uncommon and population-specific, which has contributed to the global creation of biobanks (Wei et al., 2021). One of the disorders linked to an accumulation of somatic mutations, structural variants, epigenetic variables, and changes in copy number is cancer, which frequently arises from a genetic background where hereditary cancer is more prevalent. The application of genomic sequencing in clinical setting have been made possible by advancements in sequencing technology and the creation of computational tools, supporting the therapeutic relevance of genomics to cancer treatment (Rossing et al., 2020). We report raw fastq data for hotspot regions of cancer-related 50 genes using fresh frozen breast carcinoma tissues retrieved from IMERI-FMUI Biobank collection. The data gathered from this study will help understand how breast cancer develops and forecast appropriate treatments based on somatic gene alterations that are associated with it.

## 2 Materials and methods

### 2.1 Sample collection and DNA purification

Sixteen freshly frozen breast cancer samples were collected from the IMER-FMUI Biobank, of which the majority were invasive carcinoma (Supplementary Table S1). Purified DNA was extracted from the tissues using the QIAamp DNA Mini Kit^®^ components in accordance with the manufacturer’s instructions (Qiagen Sciences). The DNA input for library preparation is 10 ng. The Nanodrop Thermoscientific 2000 instrument (ThermoFisher) was used to assess DNA purity at a 260/280 absorbance ratio, whereas the Qubit^®^ 3.0 Fluorometer and the Qubit dsDNA BR Assay Kit (Thermo Fisher Scientific) were used to determine ultimate DNA concentration. The findings are displayed in Supplementary Table S2.

### 2.2 Library preparation and sequencing

DNA libraries were created using AmpliSeq™ for the Illumina Cancer Hotspot Panel v2 (Illumina^®^, United States). This panel detects somatic variants in a total of 50 cancer-associated genes (Supplementary Table S3). The first step was amplification of target regions of the DNA sample, along with AmpliSeq™ for the Illumina Cancer Hotspot Panel v2 (Illumina^®^, United States) as well, with 17 cycles of 99°C for 15 s, 60°C for 4 min and then hold at 10°C for up to 24 h at thermal cycler. The indexes were than ligated following LIGATE program on thermal cycler (22°C for 30 min, 68°C for 5 min, 72°C for 5 min and then hold at 10°C for up to 24 h). 30 μl of the Agencourt AMPure XP beads (Beckman Coulter™, United States) was added to the mixtures to clean up the libraries.

The second amplification steps were conducted to ensure sufficient quantity for sequencing on Illumina systems. This step used 7 cycles of 98°C for 15 s, 64°C for 1 min and then hold at 10°C for up to 24 h at thermal cycler. The second cleanup was performed twice to remove high molecular-weight DNA and primer excess by using 25 μl and 60 μl of Agencourt AMPure XP beads (Beckman Coulter™, United States), respectively. The libraries were diluted to the final loading concentration at 7–9 pM and sequenced using the Illumina MiSeq platform.

## 3 Descriptive analysis

Paired-end libraries (2 × 150 bp) in fastq format were generated by the sequencing operation. Under the BioProject accession number PRJNA820526, the data sequences were submitted to the SRA. FastQC software was used to evaluate the quality of each sample’s paired-end raw readings (Andrews, 2010), and q30 Python programs were used to determine the total number of raw bases and the percentage of Q30 (Chen, 2016). Mosdepth software was used to calculate amplicon mean coverage depth, Coverage Uniformity, and on target rate (Pedersen and Quinlan, 2018).

Illumina sequencing raw read data is stored as a text file in the FASTQ format. Each sequencing read is stored in the FASTQ format on four lines of text, which provide the following information for each nucleotide: 1) identifiers, 2) nucleotide sequences, 3) “+” symbols, and 4) base quality. The first identification line includes useful data, such as the machine name, run ID, lane ID, and flow cell ID, that can be utilized to identify batch effects. The total number of reads sequenced, the GC content, and the overall base quality score are the most frequent metrics to be examined at the raw data level and are all frequently calculated by typical raw data QC programs (Sheng et al., 2017). In Table 1, we provide the descriptive details of the raw data.

**TABLE 1:** Descriptive details of the raw data.

Sample	BioSample accession number	SRA accession number	Total raw reads	Total raw bases (base-pairs)	Q30 (%)	GC content (%)	Amplicon mean coverage depth	Coverage uniformity	On target rate (%)
2017/165/mammae	SAMN27124422	SRR18574464	331102	76430999	94.9	49	24015	0.9828	96.78
2017/188/mammae	SAMN27124579	SRR18574462	313629	71977946	98.8	45	3480	0.9828	97.01
2016/114/mammae	SAMN27123254	SRR18574455	341468	77613262	96.0	44	5394	0.9828	96.79
2016/126/mammae	SAMN27123537	SRR18574454	256296	58782328	94.7	47	7384	0.9943	97.53
2016/136/mammae	SAMN27123540	SRR18574453	288351	65592639	95.6	43	4316	0.9887	95.20
2016/089/mammae	SAMN27123191	SRR18574457	269501	62131541	96.1	45	3969	0.9887	97.00
2016/083/mammae	SAMN27122511	SRR18574458	358083	82639573	95.9	45	4675	0.9943	96.74
2016/091/mammae	SAMN27123192	SRR18574456	203692	46827620	95.2	46	2161	0.9943	96.93
2015/063/mammae	SAMN27121907	SRR18574459	366344	83372684	95.1	44	4189	0.9832	96.70
2015/016/mammae	SAMN27121269	SRR18574467	325889	74670121	95.7	46	3365	0.9828	97.51
2016/151/mammae	SAMN27124383	SRR18574465	301098	68637912	95.4	44	4331	0.9782	96.55
2017/175/mammae	SAMN27124447	SRR18574463	310673	72139875	95.7	48	13169	0.9887	97.47
2015/017/mammae	SAMN27121276	SRR18574460	286654	65306734	95.7	45	2956	0.9829	97.67
2017/229/mammae	SAMN27124580	SRR18574461	297847	67899013	95.7	45	3097	0.9828	97.14
2016/145/mammae	SAMN27124350	SRR18574466	371467	87183179	95.5	49	32048	0.9828	97.50
2014/003/mammae	SAMN27121262	SRR18574468	318764	73121988	95.9	45	3678	0.9887	96.79

BioSample database: https://www.ncbi.nlm.nih.gov/biosample?Db=biosample&DbFrom=bioproject&Cmd=Link&LinkName=bioproject_biosample&LinkReadableName=BioSample&ordinalpos=1&IdsFromResult=820526

**FIGURE 1:**
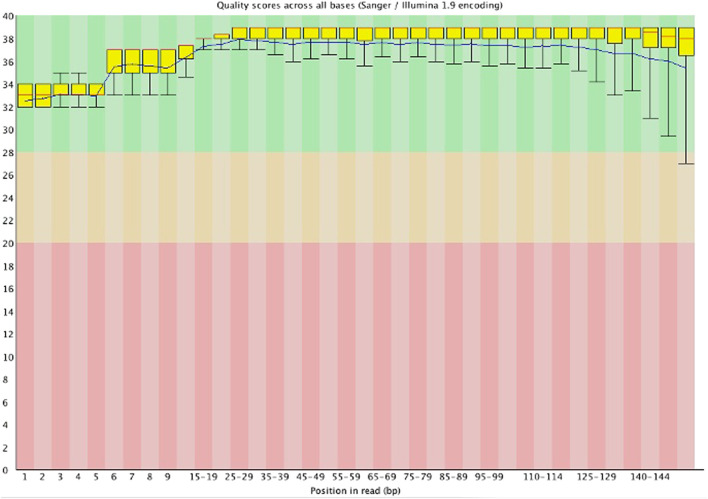
Per base sequence quality of “2016/083/mammae” sample (Generated by FastQC software).

Table 1 indicates that all the samples had a Q30 score above 90%, with the sequence quality presented from “per base sequence quality” generated by FastQC software in Figure 1. For example, for the “2016/083/mammae” sample, it showed that all the bases in the reads had a Q score above 32 (*p* error less than 0.00063), indicating high quality data produced by the illumina instrument.

## Data Availability

The datasets presented in this study can be found in online repositories. The names of the repository/repositories and accession number(s) can be found below: https://www.ncbi.nlm.nih.gov/, PRJNA820526.
