# Supplementary material for: Raw fastq data for hotspot regions of cancer-related 50 genes using fresh frozen breast carcinoma tissues obtained from IMERI-FMUI biobank collections
Source: Front Genet. 2022 Oct 24;13:973453. doi: 10.3389/fgene.2022.973453 (PMC9644235; doi:10.3389/fgene.2022.973453)
Supplement: Supplementary file 1 [file Table1.DOCX]

**Table 1.** Breast cancer sample types

| **Sample** | **Sample types** |
| --- | --- |
| 2017/165/mammae | Invasive carcinoma |
| 2017/188/mammae | Invasive carcinoma |
| 2016/114/mammae | Invasive carcinoma |
| 2016/126/mammae | Invasive carcinoma |
| 2016/136/mammae | Invasive carcinoma |
| 2016/089/mammae | Invasive carcinoma |
| 2016/083/mammae | Invasive carcinoma |
| 2016/091/mammae | Invasive carcinoma |
| 2015/063/mammae | Invasive carcinoma |
| 2015/016/mammae | Medullar carcinoma |
| 2016/151/mammae | Secretory carcinoma |
| 2017/175/mammae | Invasive carcinoma |
| 2015/017/mammae | Inflammatory carcinoma |
| 2017/229/mammae | Ductal carcinoma |
| 2016/145/mammae | No-special type |
| 2014/003/mammae | Solid papiler carcinoma |

**Table 2.** Double-stranded DNA purity and concentration

| **Sample** | **Purity (260/280)** | **Qubit 3.0 (C) (μg/mL)** |
| --- | --- | --- |
| 2017/165/mammae | 2.03 | 42.6 |
| 2017/188/mammae | 1.98 | 74.7 |
| 2016/114/mammae | 2.01 | 91.8 |
| 2016/126/mammae | 2.00 | 42.6 |
| 2016/136/mammae | 2.00 | 50.7 |
| 2016/089/mammae | 2.01 | 55.1 |
| 2016/083/mammae | 2.01 | 45.2 |
| 2016/091/mammae | 1.99 | 24.1 |
| 2015/063/mammae | 1.95 | 90 |
| 2015/016/mammae | 1.96 | 24.9 |
| 2016/151/mammae | 2.05 | 64.2 |
| 2017/175/mammae | 1.98 | 33.7 |
| 2015/017/mammae | 1.94 | 133 |
| 2017/229/mammae | 2.02 | 525 |
| 2016/145/mammae | 2.03 | 31.5 |
| 2014/003/mammae | 1.97 | 25.8 |

**Table 3**. Genes list target of AmpliSeq™ for Illumina Cancer Hotspot Panel v2

| ABL1 | EGFR | GNAS | KRAS | PTPN11 |
| --- | --- | --- | --- | --- |
| AKT1 | ERBB2 | GNAQ | MET | RB1 |
| ALK | ERBB4 | HNF1A | MLH1 | RET |
| APC | EZH2 | HRAS | MPL | SMAD4 |
| ATM | FBXW7 | IDH1 | NOTCH1 | SMARCB1 |
| BRAF | FGFR1 | JAK2 | NPM1 | SMO |
| CDH1 | FGFR2 | JAK3 | NRAS | SRC |
| CDKN2A | FGFR3 | IDH2 | PDGFRA | STK11 |
| *CSF1R* | FLT3 | KDR | PIK3CA | TP53 |
| *CTNNB1* | GNA11 | KIT | PTEN | VHL |
